# Soluble epoxide hydrolase‐derived linoleic acid diols, cerebral small vessel disease markers and white matter microstructural integrity in type 2 diabetes mellitus

**DOI:** 10.1002/alz70856_104608

**Published:** 2025-12-26

**Authors:** Si Won Ryoo, William Z. Lin, Myuri Ruthirakuhan, Yuen Yan Wong, Natasha Z. Anita, Malcolm Binns, Manuel Montero‐Odasso, Stephen R. Arnott, Carmela Tartaglia, Anthony E. Lang, Sean Symons, Robert A Hegele, Bradley J. MacIntosh, Krista L Lanctôt, Ameer Y. Taha, Sandra E. Black, Walter Swardfager

**Affiliations:** ^1^ University of Toronto, Toronto, ON, Canada; ^2^ Sunnybrook Research Institute, Toronto, ON, Canada; ^3^ University of California, San Diego, La Jolla, CA, USA; ^4^ Dalla Lana School of Public Health, University of Toronto, Toronto, ON, Canada; ^5^ Rotman Research Institute, Baycrest Academy for Research and Education, Toronto, ON, Canada; ^6^ Lawson Health Research Institute, London, ON, Canada; ^7^ Schulich School of Medicine & Dentistry, Division of Geriatric Medicine, Western University, London, ON, Canada; ^8^ Western Univeristy, London, ON, Canada; ^9^ Indoc Systems, Toronto, ON, Canada; ^10^ University Health Network, Toronto, ON, Canada; ^11^ Toronto Western Hospital, Toronto, ON, Canada; ^12^ Sunnybrook Health Sciences Centre, Toronto, ON, Canada; ^13^ Western University, London, ON, Canada; ^14^ Sunnybrook Research Institute, University of Toronto, Toronto, ON, Canada; ^15^ University of California, Davis, Davis, CA, USA; ^16^ Heart and Stroke Foundation Canadian Partnership for Stroke Recovery, Toronto, ON, Canada; ^17^ Sunnybrook Health Sciences Centre, University of Toronto, Toronto, ON, Canada; ^18^ Toronto Dementia Research Alliance, Toronto, ON, Canada

## Abstract

**Background:**

While polyunsaturated fatty acids can be metabolized into beneficial, anti‐inflammatory epoxides, they are subsequently converted to inactive, cytotoxic diols by soluble epoxide hydrolase (sEH). Increased linoleic acid (LA)‐derived diols have been associated with poorer cognitive performance in type 2 diabetes mellitus (T2DM). Furthermore, increased LA‐derived diol to epoxide ratio has been associated with greater white matter hyperintensities (WMH) in patients with stroke. However, the relationship between diols and neuroimaging measures has yet to be explored in T2DM. Here, we examined LA‐derived diols in relation to markers of cerebral small vessel disease and white matter microstructural integrity in T2DM.

**Method:**

Participants with neurodegenerative/cerebrovascular disorders recruited from the Ontario Neurodegenerative Disease Research Initiative were classified as having T2DM based on a self‐report, glycosylated hemoglobin (HbA1c) level ^3^6.5%, or antidiabetic medication use. Two unesterified LA‐derived diols (9,10‐dihydroxyoctadecamonoenoic acid [DiHOME] and 12,13‐DiHOME) were quantified via ultra‐high‐performance liquid chromatography‐mass spectrometry/mass spectrometry from serum samples collected after fasting. Perivascular spaces (PVS), WMH, and lacunar infarct volumes were quantified through structural MRI. White matter microstructural integrity was measured as fractional anisotropy (FA) and mean diffusivity (MD) using diffusion tensor imaging. Multiple linear regressions controlling for demographic factors, intracranial volume, hypertension, *APOE‐ε4* status, HDL, HbA1c, neurodegenerative diagnoses, and antidiabetic medication use, were used. Results were Bonferroni adjusted for the two diol species tested, with *p* <0.025 considered as statistically significant.

**Result:**

Among 90 T2DM participants (20.0% female, age=69.4±7.2 years, MoCA=25±3.5), 9,10‐DiHOME was associated with higher WMH (β=0.385, *p* <0.001) and lacunar infarct (β=0.283, *p* = 0.010) volumes, and higher MD (β=0.267, *p* = 0.018). The 12,13‐DiHOME was associated with higher WMH volume (β=0.339, *p* = 0.001). In exploratory regional analyses, 9,10‐DiHOME was associated with greater WMH (β=0.400, *p* <0.001) and lacunar infarct (β=0.281, *p* = 0.013) volumes, higher MD (β=0.364, *p* = 0.001), and lower FA (β=‐0.245, *p* = 0.022) in the parietal lobe. The 12,13‐DiHOME was associated with more WMH volume (β=0.352, *p* = 0.001) and higher MD (β=0.263, *p* = 0.018) in the parietal lobe.

**Conclusion:**

In diabetes, LA‐derived sEH diols were associated with greater cerebral small vessel disease pathology and white matter microstructural damage. sEH may be a potential therapeutic target to mitigate neurovascular and white matter damage in diabetes.

